# Phosphate limitation and ocean acidification co-shape phytoplankton physiology and community structure

**DOI:** 10.1038/s41467-023-38381-0

**Published:** 2023-05-10

**Authors:** Senjie Lin

**Affiliations:** Department of Marine Sciences, University of Connecticut, Groton, CT 06340 USA

**Keywords:** Marine biology, Microbial biooceanography, Marine biology

## Abstract

This Comment discusses the complexity of how ocean acidification and phosphate limitation affect phytoplankton physiologies, as well as what future research is needed to address remaining crucial questions.

## Teaser

A new study reports synergistic inhibitory effects of ocean acidification and phosphate limitation on the nitrogen-fixing capacity of a globally important cyanobacterium species. Inspired by the report, this Comment presents the complexity of how ocean acidification and phosphate limitation affect phytoplankton physiologies and species beyond nitrogen fixation and cyanobacteria, and what future research is needed to address the remaining crucial questions.

Increasing CO_2_ emission and climate change have manifold impacts on ocean primary production and carbon sequestration. One of the direct effects comes from ocean acidification due to the dissolution of ~30% of the increased CO_2_ into the ocean, whereas indirect impacts mainly stem from warming-driven ocean stratification that impedes upwelling of nutrient-rich deep waters leading to oligotrophication of the vast central ocean basin^1^. Between nitrogen and phosphate, the two major productivity-limiting nutrients, phosphate is the ‘ultimate’ limiting nutrient as it has no biogenic source, and its growth-limiting condition in the oceans is more prevalent than previously thought^2^. Nitrogen, in contrast, can be sourced from the atmosphere by diazotrophic bacteria through nitrogen fixation, which is often co-limited by phosphate and iron scarcity^2^.

**Fig. 1:**
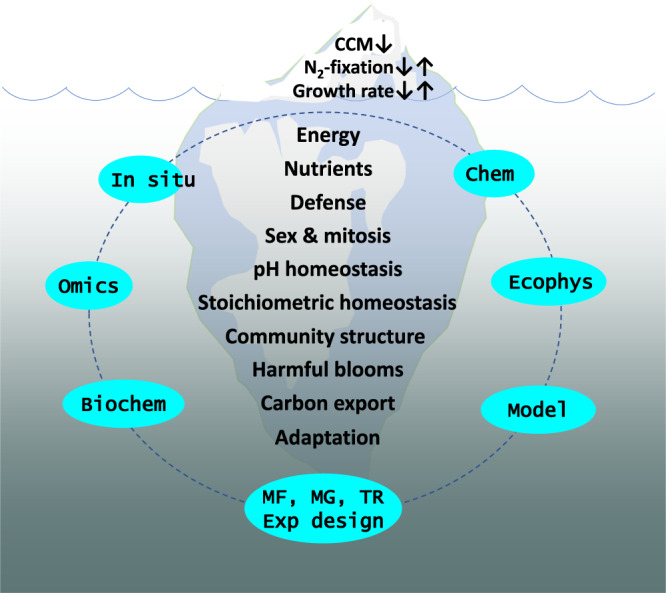
Iceberg illustration of the myriad physiological and biogeochemical processes that need to be investigated in the context of ocean acidification and phosphate limitation in phytoplankton. Among the processes shown within the iceberg, the responses of nitrogen fixation, carbon concentrating mechanism (CCM) and growth rate are relatively more investigated and understood in the context of acidification and phosphate limitation, whereas other processes shown are underexplored or poorly understood. An integrative approach (dashed orbit) is needed to effectively address outstanding questions, involving ecophysiological (Ecophys), chemical (Chem), biochemical (Biochem), multi-omics (Omics) and modeling (Model) methodologies, using in situ or laboratory studies with experimental (Exp) designs including multi-factorial (MF), multiple generations (MG), and transplant (TR) experiments. Specific questions pertaining to these processes and research approaches needed to address them are shown in Box 1.

The combination of ocean acidification and phosphorus limitation theoretically has profound impacts on phytoplankton physiologies, such as carbon fixation and nitrogen fixation. In *Nature Communications*, Zhang et al.^3^ provide robust evidence for the combined negative effects of ocean acidification and phosphorus limitation on nitrogen fixation in *Trichodesmium*, which are nitrogen-fixing cyanobacteria that significantly contribute to the global ocean nitrogen budget. Dalin Shi, the senior author of this study, and colleagues previously indicated that ocean acidification represses nitrogen fixation in *Trichodesmium*, particularly under iron limitation, which might intensify in a more acidic future ocean^4^. Contrary to the intuition that acidic conditions usually favor metal solubility, ocean acidification changes the binding characteristics of some iron ligands, resulting in lowered bioavailability of dissolved iron. The new study^3^ shows that ocean acidification exacerbates the negative effects of phosphate deficiency mainly because the cells seem to accumulate polyphosphate. Polyphosphate is a polymer of phosphate with high-energy bonds, and despite its documented roles in storing luxuriously absorbed phosphate and responding to phosphate deficiency^5^, its accumulation in acidified and P-stressed *Trichodesmium* cells suggest that it might have a role in buffering intracellular pH decreases in response to ocean acidification. Yet, this understanding creates a dilemma, because polyphosphate accumulation raises phosphorus demand, increasing phosphate deficit for metabolism. Conversely, Zhang et al.^3^ also indicate that phosphate limitation aggravates the deleterious effects of ocean acidification on *Trichodesmium* because it forces the cells to reallocate intracellular resources initially dedicated to maintaining intracellular pH homeostasis for both this function and for enhancing phosphate uptake. Consequently, nitrogen fixation and carbon fixation cost more phosphate and are thus markedly repressed under ocean acidification. Based on the Community Earth System Model run under the IPCC RCP 8.5 scenario, the authors find that ocean acidification combined with phosphate limitation can reduce nitrogen fixation in the ocean by 22.8 TgN yr^−1^ in the period of 2081–2100 compared to the 11.3 TgN yr^−1^ reduction by acidification alone. These findings shed light on the complexity of the interactive effects on phytoplankton of climate change with the marine chemical environment, but they only represent a tip of the ‘Ocean acidification–Phosphorus limitation’ iceberg (Fig. 1). In this Comment, recent advances in relevant research areas will be discussed to demonstrate the complexities of dual ocean acidification–phosphorus limitation stress on phytoplankton physiology and community structure beyond cyanobacteria and nitrogen fixation.

## Phytoplankton physiology and ocean acidification

**Fig. 2:**
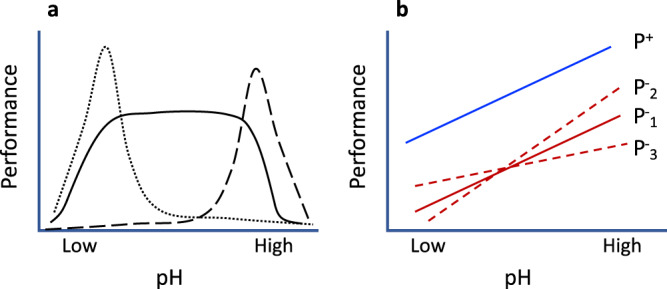
Illustration of different types of responses to ocean acidification alone or in tandem with phosphate limitation. **a** Response to ocean acidification alone. Generalists have a broad pH niche breadth (solid line) whereas low-pH specialists (dotted line) and high-pH specialists (dashed line) are adapted to low and high pH, respectively. **b** Response to combined ocean acidification and phosphate limitation. Reaction norms of performance under P-replete (P^+^) and P-depleted (P^-^) conditions. P^+^ and P^-^_1_ (solid lines, with the same slope) indicate additive effects of ocean acidification and P limitation, that is, with no interactive effects between the two stressors. P^+^ and P^-^_2_ (greater slope in P^-^_2_) depict synergistic interactive effects of ocean acidification and P limitation. P^+^ and P^-^_3_ (smaller slope in P^-^_3_) show an antagonistic interaction between ocean acidification and phosphate limitation.

Ocean acidification effects vary with phytoplankton species depending on their distinct genetics and physiologies related to energy, nutrition, and defense, which together regulate population growth. It has been shown that ocean acidification promotes growth of some species, inhibits the growth of others, and has little effect on yet other species^1,6^. These different ecotypes are low-pH specialists, high-pH specialists, or pH generalists, respectively (Fig. 2a). Ocean acidification can benefit species that usually rely on energetically expensive carbon concentrating mechanisms (CCM) to obtain adequate CO_2_ for photosynthesis (Fig. 1). The CO_2_-fixing enzyme Rubisco is very inefficient, with a half-saturation constant of ~100 µM or higher while CO_2_ concentration in the surface ocean is only ~10 µM^7^. Therefore, Rubisco is less than half-saturated under current CO_2_ levels^8^. Except for some terrestrial green algae and some freshwater red algae^8^, most algae have evolved CCM so that cells can take up the abundant bicarbonate in the ocean (~2000 µM) through bicarbonate transporters and convert it to CO_2_ via carbonic anhydrase. CCM operations cost energy. Presumably, at elevated CO_2_ (ocean acidification), the demand for CCM and the associated energy required in species that rely on CCM will be lowered, although the magnitude of the benefit is generally unclear but may be limited in lineages such as diatoms^9^. However, ocean acidification also causes stress to phytoplankton cells as it perturbs intracellular pH homeostasis, which imposes energetic costs to maintain.

Proton pump rhodopsin (PPR), which occurs in many but not all phytoplankton species, can potentially help maintain pH homeostasis in PPR-containing species. With all-trans retinal as the light receptor, PPR uses photoenergy to pump protons across cell membranes, resulting in ATP production^10^. Relying on only one protein, in contrast to the photosynthetic apparatus that requires hundreds of proteins, PPR is a highly efficient energy harvesting system. Interestingly, PPR may not only provide energy in form of ATP, but also directly remove excess protons from inside cells to facilitate pH homeostasis. Evidence of the latter function exists in bacterioplankton PPR^11^. Furthermore, PPR-derived energy may even ameliorate phosphorus stress. Studies have shown the dinoflagellate *Prorocentrum shikokuense* upregulates PPR expression under phosphorus limitation^12^ and is prone to form blooms in phosphorus-limited waters while actively expressing PPR^13^. Hence, species equipped with CCM and PPR could perform better than species without these apparatuses in acidified oceans, but the net outcome generally depends on the offset between the cost imposed by ocean acidification and the benefit provided by these and potentially many other mechanisms.

Phytoplankton species also differ in their capacities and mechanisms to cope with phosphate limitation, which is quite prevalent in the global ocean^14^ and will likely be intensified by climate change. Under phosphate deficiency, phytoplankton usually reduce phosphorus demand by substituting phospholipids with sulfur- or nitrogen-containing lipids^12,15^. More importantly, phytoplankton most commonly use alkaline phosphatase but also other hydrolases to scavenge dissolved organic phosphorus (DOP) from the water column for phosphorus^16^. However, alkaline phosphatase activity is sensitive to pH changes. While a basic pH is generally preferrable to alkaline phosphatase, its optimal pH varies between species, with some species potentially benefiting from ocean acidification for scavenging DOP, as shown in a preprint article by Guo et al.^17^ (see Fig. 1 of ref. ^17^). Furthermore, some phytoplankton hydrolyze DOP using acid phosphatase or nucleotidase or take up DOP without hydrolysis^16,18^, but how these strategies are affected by ocean acidification is unclear. In addition, many phytoplankton species are mixotrophic and utilize algal prey via phagocytosis when nutrients are depleted^19^. As the digestion of prey inside the lysosome-fused phagosome requires low pH, acidification inside the phagosome might enhance this mode of nutrition, but how ocean acidification may impact mixotrophy remains to be investigated. PPR can potentially pump H^+^ into food vacuoles in mixotrophic species to enhance phagotrophic activity, as suggested by PPR co-localization with food vacuole membranes in a heterotrophic dinoflagellate^20^. Overall, ocean acidification will differentially influence the success of different phytoplankton species to cope with phosphate limitation through impacting their capacity to utilize DOP, to perform phagotrophy or to harness supplementary energy.

In some phytoplankton species, both ocean acidification and phosphorus stress can cause shifts of intracellular resources towards defense such as producing more toxins. For instance, phosphorus limitation-induced toxin production in the dinoflagellate *Karlodinium veneficum* could be stimulated by elevated CO_2_^21^, indicating synergistic effects that will be discussed further below. Toxin production is one of the common strategies in phytoplankton to deter grazing, so there is potential for a strengthened defense to partially offset the population decline caused by slower growth under ocean acidification and/or phosphate limitation.

Besides their independent effects, ocean acidification and phosphorus limitation can interact while acting on phytoplankton, and the combined effect can be very complex. The interactive effects can be additive, synergistic or antagonistic (Fig. 2b), as previously demonstrated for ocean acidification and nitrogen limitation or other environmental variables^1^. For instance, an ocean acidification–nitrogen limitation interaction was found to promote growth in the diatom *Chaetoceros didymus*, whereas ocean acidification alone showed no effect and nitrogen limitation depressed growth^1^. Apart from these synergistic effects, antagonistic interactions have been reported in some phytoplankton. For example, elevated *p*CO_2_ partially mitigated the inhibitory effects of UV radiation^1^ or nitrogen limitation^22^ on phytoplankton growth. These indicate that increasing CO_2_ can indeed provide benefits to counteract the detrimental effects of UV radiation or nitrogen limitation, although the underpinning mechanisms are obscure.

Another layer of complexity comes from the interaction of these environmental factors with phytoplankton genetics. If stressful environmental conditions are constant for a sufficiently long period, lineages will generally be expected to adapt to produce a fixed phenotype with high fitness under those environmental conditions, but potentially lower fitness when environmental conditions return to the previous state. However, if conditions have been variable, lineages may have been selected to be phenotypically plastic, producing a different phenotype depending on the combination of pH and phosphorus through changes in epigenetic regulations and gene expression. As such, plasticity allows the phenotype and fitness to rebound once less stressful environmental conditions return.

## Phytoplankton community and ecological implications

Clearly, the impacts of ocean acidification and phosphorus limitation reach far beyond nitrogen fixation and cyanobacteria. Furthermore, the effects can manifest at different scales of organization, from molecular to population and community levels. Due to the differential responses associated with distinct genetics and physiologies of different species, the ocean acidification–phosphorus limitation dual-stressor will likely lead to disparate ecological outcomes in future oceans under climate change^6^. One potential outcome is altering community-level performance; for example, productivity and other ecosystem services^23^. Alternatively, the community may be resilient to the stressors and there are no detectable changes of community productivity or abundance under stress. For example, in the Arctic, a phytoplankton community treated with elevated *p*CO_2_ in an on-deck incubation maintained stable performance (bulk net productivity) under acidification treatment^24^. The community insensitivity might be due to the stability of species composition reflecting broad niche width of the constituent species or strain sorting coupled with high intraspecific diversity for the constituent species. Alternatively, it could be due to the occurrence of functionally redundant species, whose shifts in response to stress caused no performance change. Strain sorting by ocean acidification has been shown in the globally distributed prasinophyte *Ostreococcus*^25^. While strain sorting or functionally redundant species shuffling may not change net community primary productivity, they could significantly change trophic linkages and other crucial ecological functions. Consequently, the effects in both scenarios could reach up in the food chain to impact fisheries and ecosystem function.

Harmful algal blooms are an important example of community responses to phosphate limitation coupled with acidification. As illustrated in Fig. 2a, the climate-stress conditions may suppress the growth of most constituent species (generalists and most specialists) in the community while allowing opportunistic, stress-adapted species (specialists) to proliferate and form blooms. A plethora of factors are potentially responsible for a harmful algal bloom, but there is evidence that their frequency, number of causative species, and ecological devastation have increased due to climate change^26^. Ecosystem-level effects can also manifest in carbon biogeochemistry, as phosphorus limitation has been shown to enhance carbon export efficiency in some species^27^ but cause no change in others^28^.

Many questions remain to be addressed regarding the effects and underlying mechanisms of ocean acidification and phosphate limitation in phytoplankton. Among others, questions that can be immediately addressed pertain to the metabolic processes of energy and nutrient acquisition, defense against grazing and microbial attacks, mode of life history (sexual reproduction and mitosis), homeostasis of intracellular pH, elemental stoichiometry, and ecological processes of community structure, harmful algal blooms, biogeochemical process of carbon export, and evolutionary processes of adaptation (Fig. 1, Box 1). PPR potentially plays roles in several parts of the energy and nutrient acquisition processes and pH homeostasis, and an in-depth inquiry is warranted. To decipher the mechanistic processes and accurately model ecological consequences, future research should adopt more holistic and interdisciplinary approaches (Box 1). Discriminating different strategies by which phytoplankton respond to ocean acidification and phosphate limitation calls for multi-generation and transplant experiments. Laboratory experiments should also be complemented by field in situ studies, and single-species experiments should be supplemented with whole-assemblage investigation. Physiological measurements should be accompanied by multi-omics profiling, and observations should be extended by modeling. Only then can many fundamental questions be properly addressed. The exciting work by Zhang et al.^3^ opens a window to a wide horizon where many new studies and novel insights about the effects of ocean acidification and phosphorus limitation on phytoplankton community structure and function, as well as carbon and nitrogen biogeochemical cycles, will emerge.

Box 1 Major questions and approaches to address them

**Table:** 

Question	Approach
How much energetic benefit can phytoplankton species with carbon-concentrating mechanisms (CCM) gain from ocean acidification?	Compare photosynthetic and growth rates of CCM-containing and CCM-absent (or blocked) strains grown under normal and acidified conditions.
Does proton pump rhodopsin (PPR) provide energy needed for PPR-containing species to cope with stress of acidification?	Compare cellular chlorophylls and rhodopsin contents and their ratios in PPR-containing species under normal and acidified conditions.
How will ocean acidification impact alkaline phosphate (AP)-dependent or AP-independent utilization of dissolved organic phosphate (DOP)?	Compare DOP utilization efficiencies between AP-dependent and AP-independent strains under normal and acidified conditions; investigate AP activities and gene expression under those conditions using biochemical and omics approaches.
How will ocean acidification promote heterotrophy or mixotrophy in protists?	Compare grazing and growth rates of mixotrophic species under normal and acidified conditions; measure intracellular and food vacuolar pH.
How will ocean acidification and phosphate limitation affect the ability of phytoplankton to defend against grazing and microbial attacks?	Examine growth and molecular responses of phytoplankton co-cultured with zooplankton and microbes under factorial design with acidification and phosphate limitation; measure toxin production if appropriate; conduct omics analyses to detect defense-related regulatory mechanisms.
How will ocean acidification alone or tandem with phosphorus limitation influence sexual and asexual reproduction?	Use multi-factorial experimental design for phytoplankton and examine cell cycle and sexual reproduction using physiological, microscopic, and omics analyses.
Does PPR serve to buffer intracellular pH variations by pumping out protons, or alleviate stress of phosphate limitation, or both?	Use multi-factorial experimental design on PPR-functioning and PPR-inhibited (or disrupted) cultures; measure growth rate and intracellular pH; conduct omics analyses to find metabolic evidence of which role PPR may play.
What functions do polyphosphate play in coping with acidification and phosphate limitation: to buffer acidity, serve as phosphate source, or relieve stress?	Investigate how phytoplankton utilize polyphosphate alone or in combination with phosphate under acidification alone or in combination with phosphate deficiency. Measure intracellular pH and analyse omics profiles to understand the roles of polyphosphate and underlying mechanisms.
How will ocean acidification and phosphate limitation impact the N:P stoichiometric homeostasis?	Use multi-factorial experimental design with different N:P regimes, measure cellular N and P contents and their molar ratios in phytoplankton; conduct omics analyses and gene knockouts to pinpoint regulators involved.
How will acidification and phosphate limitation alter diversity and community structure of phytoplankton?	Use co-cultures or natural-assemblage mesocosms with acidified and phosphate-limited conditions or conduct in situ fieldwork; carry out physiological measurements of species-specific performance, molecular analyses for community structure, and omics profiling of metabolic responses.
How will acidification and phosphate limitation affect the likelihood of harmful algal bloom (HAB) outbreaks?	Use co-cultures or natural-assemblage mesocosms with acidified and phosphate-limited conditions or conduct in situ fieldwork; carry out physiological measurements of species-specific performance, molecular analyses for community structure, and omics profiling of metabolic responses; compare HAB species with others.
How will ocean acidification alone or in tandem with phosphate limitation influence phytoplankton-mediated carbon export?	Measure sinking rates and carbon contents of phytoplankton cells grown under normal, acidified, phosphate limited, and tandem acidified-phosphate stress conditions.
Are phytoplankton physiologically plastic to ocean acidification? What are the mechanisms underpinning responses to acidification within and across generations? How can these mechanisms evolve in future acidified and phosphate-limited oceans?	Use multi-generation and transplant experiments; measure physiological parameters; use omics approaches coupled with functional genetic (for example, CRISPR/Cas9 genome editing) approaches to identify genetic bases.
